# Dual Burst and Sustained Release of *p*-Coumaric Acid from Shape Memory Polymer Foams for Polymicrobial Infection Prevention in Trauma-Related Hemorrhagic Wounds

**DOI:** 10.1021/acsami.3c04392

**Published:** 2023-05-15

**Authors:** Changling Du, David Anthony Fikhman, Devanand Persaud, Mary Beth Browning Monroe

**Affiliations:** Department of Biomedical and Chemical Engineering, Bioinspired Institute for Material and Living Systems, Syracuse University, Syracuse, New York 13244, United States

**Keywords:** phenolic acid, antimicrobial, antibiofilm, shape memory polymer, trauma, wound healing, polymicrobial infection

## Abstract

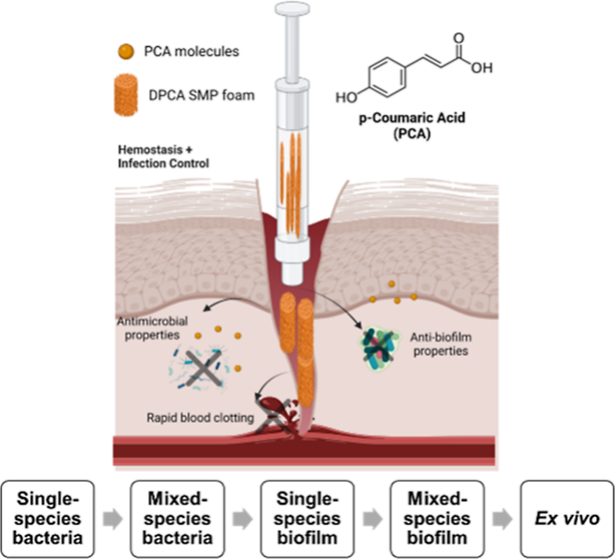

Hemorrhage is the primary cause of trauma-related death. Of patients that survive, polymicrobial infection occurs in 39% of traumatic wounds within a week of injury. Moreover, traumatic wounds are susceptible to hospital-acquired and drug-resistant bacterial infections. Thus, hemostatic dressings with antimicrobial properties could reduce morbidity and mortality to enhance traumatic wound healing. To that end, *p*-coumaric acid (PCA) was incorporated into hemostatic shape memory polymer foams by two mechanisms (chemical and physical) to produce dual PCA (DPCA) foams. DPCA foams demonstrated excellent antimicrobial and antibiofilm properties against native *Escherichia coli*, *Staphylococcus aureus*, and *Staphylococcus epidermidis*; co-cultures of *E. coli* and *S. aureus*; and drug-resistant *S. aureus* and *S. epidermidis* at short (1 h) and long (7 days) time points. Resistance against biofilm formation on the sample surfaces was also observed. In *ex vivo* experiments in a porcine skin wound model, DPCA foams exhibited similarly high antimicrobial properties as those observed *in vitro*, indicating that PCA was released from the DPCA foam to successfully inhibit bacterial growth. DPCA foams consistently showed improved antimicrobial properties relative to those of clinical control foams containing silver nanoparticles (AgNPs) against single and mixed species bacteria, single and mixed species biofilms, and bacteria in the *ex vivo* wound model. This system could allow for physically incorporated PCA to first be released into traumatic wounds directly after application for instant wound disinfection. Then, more tightly tethered PCA can be continuously released into the wound for up to 7 days to kill additional bacteria and protect against biofilms.

## 1 Introduction

Uncontrolled bleeding is the leading cause of traumatic injury-related pre-hospital death, and it accounts for 40% of deaths in the first 24 h of injury.^1−5^ Early bleeding control has proven effective in saving lives in the military, medical, and civilian environments. The most common hemostatic agent available on the market today is a combination of gauze, such as Quick Clot Combat Gauze (Teleflex, PA, USA), and tourniquets. Unfortunately, this combination cannot be used in non-compressible torso and junctional (neck, axilla, and groin) bleeding wounds, which account for 48 and 21% of combat casualties, respectively.^6^

In response, newer hemostatic dressings have been developed in recent years. XSTAT (RevMedx, OR, USA) is an FDA-approved hemostatic dressing. XSTAT comprises ∼92 mini cellulose compressed sponges that can be inserted into the wound through an injector, where they expand rapidly to apply pressure to wound walls and stop bleeding. However, the mean time for surgical removal of XSTAT sponges is 22 times longer than that of gauze, complicating their use.^7^ The newer XSTAT 30 has a pellet-containing radiopaque bag to simplify individual pellet retrieval. However, Bonanno *et al.* reported that this design reduced the overall survival rate, which may be due to changes in liquid absorption and expansion of sponge pellets.^8^ In general, XSTAT is effective, but according to Tactical Combat Casualty Care (TCCC) guidelines,^9^ it still has drawbacks, including the following: (1) sponges need to be removed within 4 h of application per the FDA clearance letter;^9−11^ (2) one or more of the mini-sponges has potential for occlusion of the carotid or jugular vessels, so radiological clearance must be employed after removal of sponges;^12^ and (3) one wound typically requires three XSTAT dressings, which costs approximately $1000.^9,13,14^ Other dressings are in development, but in general, none have emerged as both safe and effective for use in noncompressible wounds.

To provide an improved hemostatic dressing material, thermally induced shape memory polymer (SMP) foams present an ideal strategy. SMP foams are a class of smart materials that can return from a deformed secondary shape to a primary shape after exposure to a stimulus, such as heating. Previously, a thermoset polyurethane foam system was developed.^15−17^ Under dry conditions up to ∼50 °C, these SMP foams maintain a compressed shape for easy storage and deep wound delivery. Then, when the foams are exposed to water in body temperature blood, they expand to their primary shape within ∼2 min to fill the wound and induce rapid blood clotting. SMP foams have been pursued for use in hemostatic applications due to their biocompatibility, hemocompatibility, and rapid clotting capabilities.^18−21^ In a noncompressible Grade V liver injury model in swine, SMP foams reduced blood loss and active bleeding time and significantly increased the 6 h survival rate compared with XSTAT and Quick Clot Combat Gauze-treated wounds.^22^ Beyond their hemostatic capabilities, SMP foams also have highly tunable chemistry that enables incorporation of additional functionalities for healing.

A major concern in current commercially available hemostatic devices is that they do not include antimicrobial function. Of patients that survive early hemorrhagic shock in the pre-hospital or emergency environments, approximately 39% develop one or more types of infection within the first week after injury.^23^ Beyond contamination during wounding and/or treatment, healthcare-associated infections (HAIs) are critical factors in the survival of trauma patients during hospitalization. According to a Centers for Disease Control and Prevention (CDC) prevalence survey released in 2014 involving 11,292 patients in 183 hospitals in the U.S., about 4% of hospitalized patients had at least one HAI.^24^ Thus, an ideal hemostatic dressing strategy aids in preventing short and long-term infectious complications.

When selecting potential antimicrobial targets for incorporation into hemostatic dressings, antibiotic-resistant infections are an important consideration. The first strain of methicillin-resistant *Staphylococcus aureus* (MRSA) was discovered in the United Kingdom in 1962 and in the United States in 1986.^25^ Since then, infections with MRSA and other drug-resistant bacteria have spread globally in response to antibiotic overuse. The World Health Organization’s director, Dr. Margaret Chan, said that the world is heading toward a post-antibiotic era in 2011; and antibiotic resistance has continued to rise since that time.^26^ Thus, we need new antimicrobials to fight drug-resistant bacteria, and non-drug-based natural antimicrobials provide excellent candidates with less selection pressure than traditional antibiotics. However, these natural antimicrobials must still be highly effective at preventing and eliminating infections to reduce biofilm formation that can contribute to chronic wound development.

In terms of case physiology, wound infection is an immune imbalance caused by bacteria in the wound. This imbalance results in an environment that favors bacterial proliferation rather than tissue regeneration. Therefore, antimicrobial wound dressings that provide a bacteriostatic environment are essential for accelerating the healing process. On this basis, wound dressings can be classified in terms of antiseptic active ingredients.^7,27−32^ Antiseptic active ingredients mainly include antibiotics; physical antiseptics, such as silver^33^ and polyhexanide; antimicrobial enzymes, such as lysozyme;^34^ and chemical antiseptics, such as chlorhexidine. In addition, honey is also employed for antiseptic wound healing, with examples including Revamil and Manuka.^35^

The main antimicrobial properties of honey are derived from phenolic compounds.^36^ Plant phenolics are the broadest secondary metabolites of plants,^37^ which present excellent antioxidant,^37−41^ anti-inflammatory,^42−44^ anticancer,^45−48^ and antimicrobial properties.^42,49−55^ As early as 1958, an article in *Nature* reported on a phenolic compound for its antibacterial properties against *Escherichia coli*.^56^ Phenolic acids (PAs) are a subclass of plant phenolics with antimicrobial properties and efficacy against mutidrug-resistant organisms (MDROs).^52−54,57^ Our previous works demonstrated that a library of PAs have antimicrobial activity against *E. coli*, native and drug-resistant *S. aureus*, and native drug-resistant *Staphylococcus epidermidis* (*S. epidermidis*).^55^ Based on that work, we selected three PAs and introduced them chemically into SMP foams.^18^ The resulting materials had improved antimicrobial properties while maintaining cytocompatibility and platelet activation capabilities. Of these three materials, the *p*-coumaric acid (PCA) foam demonstrated the most potent antimicrobial properties, but it did not completely eliminate the surrounding bacteria. Others have shown that PCA can inhibit the growth of various bacterial pathogens by increasing the permeability of bacterial outer and plasma membranes, leading to the destruction of the bacterial cell.^58^ In addition, PCA can bind to the bacterial DNA double helix to inhibit cellular functions and kill bacteria by dual bactericidal mechanisms.^58^ PCA also exhibits resistance to biofilm formation.^59^ Thus, PCA provides a potential antimicrobial agent for improved infection control capabilities in SMP foams.

**Figure 1:**
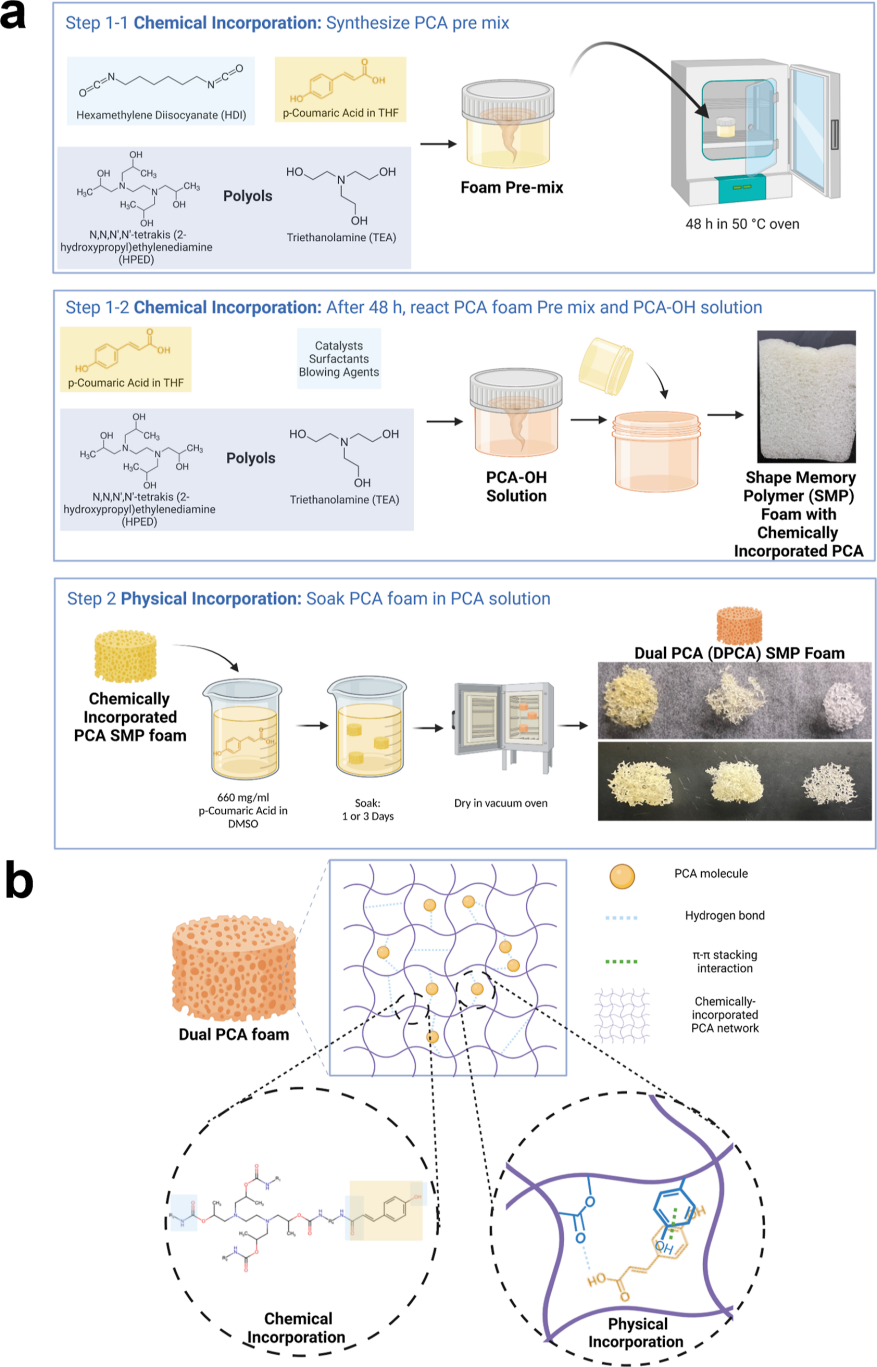
Design strategy of dual *p*-coumaric acid (DPCA) SMP foams. (a) Synthesis process of DPCA SMP foams *via* chemical incorporation (step 1) and physical incorporation (step 2). (b) Molecular structure of DPCA SMP foam. Created with BioRender.com.

In this study, we aimed to increase the content of PCA in the SMP foam network to improve antimicrobial and antibiofilm properties through a dual incorporation mechanism to form dual PCA (DPCA) foams, as shown in Figure 1a. Namely, PCA was chemically introduced into the SMP foam network during synthesis, followed by physical incorporation of PCA post-fabrication. For chemical incorporation, PCA carboxylic acid groups were reacted with isocyanates used to form the polyurethane backbone to provide amide bond tethers to the SMP foam network. During physical incorporation, PCA was coated on the surface of SMP foams *via* hydrogen bonding and π–π stacking interactions. We hypothesize that when a DPCA foam is placed into a wound, the hydrogen bonding and π–π stacking interactions can be rapidly broken by water in the body to provide a burst release of PCA that disinfects the wound during the pre-hospital period. Then, chemically incorporated PCA exhibits a sustained presentation in the wound to enable longer term antimicrobial prevention and biofilm inhibition during hospitalization, as shown in Figure 1b.

## 2 Materials and Methods

### 2.1 Materials

All chemicals were purchased from Fisher Scientific (Hampton, NH, USA) unless otherwise specified. *p*-coumaric acid (PCA) was purchased from TCI America Inc (Portland, OR, USA). NIH 3T3 mouse fibroblasts and bacteria strains were purchased from ATCC (Manassas, VA, USA).

### 2.2 Synthesis of *p*-Coumaric Acid-Containing Foams

Dual PCA incorporation contains two steps for chemical and physical incorporation, as shown in Figure 1a. Chemically incorporated PCA foam was synthesized using the previously described method.^18,60^ Briefly, PCA, polyols (triethanol amine and hydroxypropyl ethylene diamine), and excess hexamethylene diisocyanate (HDI) were mixed at 50 °C for 48 h to form an isocyanate (NCO) pre-polymer. A COOH/OH solution was prepared with remaining PCA and polyols to provide a 1:1 ratio of COOH + OH/NCO groups. Catalysts (BL22 and T131), surfactant (EPH190), and a blowing agent (deionized water) were added. The NCO pre-polymer was reacted with the COOH/OH solution at 50 °C to form a PCA-containing SMP foam. After that, the PCA SMP foam was immersed in a 660 mg/mL PCA solution in anhydrous dimethyl sulfoxide (DMSO) for 1 or 3 days to enable physical incorporation of PCA into the polyurethane network *via* hydrogen bonding and π–π stacking. All dual PCA (DPCA) foams were dried at 50 °C under vacuum to remove excess DMSO. For analysis, a control foam was synthesized with no PCA. The chemically incorporated PCA foam was compared with DPCA foams that had been soaked in PCA solutions for 1 day and 3 days to include both chemically and physically incorporated PCA.

### 2.3 DPCA Foam Characterization

#### 2.3.1 Surface Chemistry

The surface chemistry of foam samples was analyzed using attenuated total reflectance Fourier transform infrared (FTIR) spectroscopy (Nicolet i70 Spectrometer, Fisher Scientific, Waltham, MA, USA) at 0.8 cm^–1^ resolution.

#### 2.3.2 Foam Thermal Transitions

The glass transition temperature (Tg) of foam samples (*n* = 3) was analyzed using a Q-200 differential scanning calorimeter (TA Instruments, Inc., New Castle, DE). Foam samples were cut into 3–5 mg pieces. Samples were placed into a T_zero_ aluminum pan and covered with an aluminum lid (DSC Consumables, Inc., Austin, MN). Samples were subjected to the following program: (1) equilibrated at −40 °C for 2 min; (2) heated to 120 °C at 10 °C per minute; (3) equilibrated at 120 °C for 2 min; (4) cooled to −40 °C at −10 °C per minute; (5) equilibrated at −40 °C for 2 min, and (6) heated again to 120 °C at 10 °C per minute in a second cycle. The Tg of samples was analyzed as the endothermic inflection point of the DSC thermogram in the second heating cycle by TA instrument software (TA Instruments, Inc., New Castle, DE, USA).

#### 2.3.3 Pore Structure

Foam samples were cut into 1 mm thick disks using a biopsy punch (Sklar Instruments, PA, USA) and fixed with double-sided tape onto a sample holder. Subsequently, foam samples were coated with Au for 45 s using a sputter-coater (Denton Vacuum Desk V, Moorestown, USA) and imaged using a Jeol NeoScope JCM-5000 scanning electron microscope (Nikon Instruments, Inc., Melville, NY, USA).

#### 2.3.4 PCA Release

Foam samples (*n* = 3) were cut into ∼20 mg pieces. Samples were placed in separate sealed vials with 5 mL of PBS:DMSO (3:2) solution in a 37 °C incubator. Sample solutions were collected at 1 h, and then each 24 h up to 7 days, with fresh 5 mL solution added at each time point. Sample solutions were placed into a black wall cuvette to measure absorbance using a Cary 60 UV–vis spectrophotometer (Agilent Technologies, Santa Clara, CA, USA). The PCA concentrations were quantified by comparison with PCA standard curves with varying PCA concentrations from 2 to 18 μg/mL.

#### 2.3.5 Volume Recovery

Foam samples (*n* = 3) were cut into 10 mm length and 3 mm diameter cylinders. Samples were heated to 100 °C for 1 h, and the volume of the samples was measured using a digital caliper. Samples were crimped into a 2 mm diameter cylinder for 5 min at room temperature in a radial compression crimper (Blockwise Engineering LLC, Temp, AZ) to execute the programming process. Samples were removed from the crimper after cooling, measured using a digital caliper, and left overnight. To check shape fixity, programmed samples were measured using a digital caliper. A 0.5 mm diameter nickel-titanium wire (NDC, Fremont, USA) was threaded through the samples to hold them in place above a metal pan. Subsequently, samples were immersed in a 37 °C water bath and photographed every 10 s over 5 min *via* GoPro (Woodman Labs, Inc., CA, USA). Foam sample volumes in each image were measured *via* ImageJ software (NIH, Bethesda, USA). The percent volume recovery of the samples was calculated using the following equation
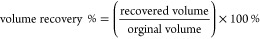


### 2.4 Cytocompatibility

Mouse embryonic NIH/3T3 cells were cultured in Dulbecco’s modified eagle medium media (DMEM, Gibco, Thermo Fisher Scientific, Waltham, MA, USA) with 10% heated-inactivated fetal bovine serum (FBS) and 1% penicillin–streptomycin (PS) in a 37 °C/5% CO_2_ incubator. Cells were used between passages 2 and 4. Relative cell numbers over time were analyzed using an Alamar Blue assay. Briefly, 3T3 cells were seeded in 24 well plates in DMEM overnight. Samples were cut into 5 mg pieces (*n* = 3) and placed into Transwell inserts above the cells in the 24-well plate. Control foams without PCA were used as the positive (cytocompatible) control group (*n* = 3). Every 24 h over 7 days, the Transwell inserts with samples were removed from the 24 well plates. The media was removed, and 600 μL of Alamar Blue cell viability reagent (Invitrogen, MA, USA) was added. Cells were incubated with the reagent for 2 h, and then 100 μL of the reagent solution was transferred into a 96-well plate from the 24-well plate. All wells of the 24 well plates were washed twice with sterile PBS and fresh DMEM was added. Transwell inserts with samples were returned to the original 24-well plate with cells in the incubator. Meanwhile, the 96-well plate was placed in a plate reader (FLx800, Bio-Teak Instrument Inc., VY, USA) to measure fluorescence intensity with an excitation of 530 nm and emission of 590 nm. Cell viability was measured as
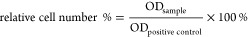
OD_sample_ is the optical density of the sample and OD_positive control_ is the optical density of unmodified SMP foam positive controls.

### 2.5 Antimicrobial Characterization

#### 2.5.1 Preparation of Single-Species and Mixed-Species Bacteria Strains

*Escherichia coli* (*E. coli*, ATCC 9637, native strain), *Staphylococcus aureus* (*S. aureus*, ATCC 51153, native), *Staphylococcus epidermidis* (*S. epidermidis*, ATCC 12228, native), drug-resistant *S. aureus* (DR *S. aureus*, ATCC 43300, drug-resistant strain), and drug-resistant *S. epidermidis* (DR *S. epidermidis*, ATCC 700566, drug-resistant strain) were used to test the antimicrobial properties of samples. Before antimicrobial testing of single species strains, bacteria were grown in 5 mL of sterile fresh Luria–Bertani (LB) broth for ∼16 h at 37 °C in an incubator. The next day, 1 mL was removed from the 5 mL bacteria solution and added to 9 mL of fresh LB. Bacteria were incubated at 37 °C until they reached the logarithmic growth period, at which an optical density at an absorbance of 600 nm (OD_600_) equaled either 0.4 for biofilm formation analysis or 0.6 for antimicrobial testing of bacteria in solution, as confirmed using a plate reader.^55^ Before testing of mixed-species bacteria, *E. coli* and *S. aureus* were cultured separately in individual tubes. After both strains had the same optical density (OD_600_ = 0.4 or 0.6 for biofilm or solution testing, respectively), *E. coli* and *S. aureus* were mixed in a 1:2 ratio.

#### 2.5.2 Short-Term Antimicrobial Protection

The quantification method of antimicrobial properties was reported in our previous article^18^ and by Shatalin *et al.*(61) Briefly, samples were cut into 5 mg pieces (*n* = 3) and sterilized by ultraviolet light. Samples were placed into a sterile 96-well plate with 100 μL of bacterial solution (OD_600_ = 0.6) to culture for 1 h at 37 °C with shaking. All bacterial solutions were diluted by 10^8^ in fresh LB. Subsequently, 10 μL of the diluted solution (*n* = 3) were drop plated onto an LB-agar plate to culture for 18 h in a 37 °C incubator. Images were obtained of each drop area after 18 h. The colony-forming units (CFUs) were quantified using ImageJ software. QuickClot Combat Gauze and a silver nanoparticle (AgNPs) containing polyurethane foam dressing (AREZA MEDICAL, TX, USA) served as clinical controls.

#### 2.5.3 Long-Term Antimicrobial Protection

Samples were cut into 5 mg pieces (*n* = 3) and sterilized by UV light. Samples were placed in a 96-well plate with 150 μL of bacteria solution (OD_600_ = 0.6, diluted by 100 times) to incubate at 37 °C. In each sample well, 50 μL of fresh LB was applied and 200 μL of sterile PBS was applied to the surrounding empty wells to reduce bacterial solution evaporation. A bacteria control contained bacteria solution without samples, and a LB control included only 150 μL of LB broth with no added bacteria. Every 24 h over 7 days, 100 μL of solution was transferred from each well into a new 96-well plate and placed in a plate reader to analyze the OD_600_. After reading, 100 μL of sample solution was returned to the samples in the original 96 well-plate in the incubator. Bacterial growth inhibition was measured as
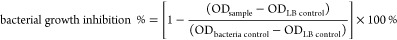


For mixed species characterization, 10 μL or 50 μL of sample solutions with varied dilution concentrations were drop plated onto an LB-agar plate on the seventh day to the culture and incubated at 37 °C for 18 h. Images were obtained, and CFUs were measured using ImageJ software as demonstrated in the Supporting Information (Figure S1).

#### 2.5.4 Anti-Biofilm Properties

Methods for visualization of biofilms were modified from the reported methods of Li *et al.*(62) and Ren *et al.*(63) Samples were cut into 8 mm diameter and 2 mm height cylinders (*n* = 3) and sterilized by UV light. Samples were placed into two 24-well plates with 400 μL of bacteria solution (OD_600_ = 0.4) in a 37 °C incubator for 24 h and 48 h. At 24 and 48 h, samples were washed by immersing in sterile PBS to remove free bacteria and then fixed with 2.5% glutaraldehyde for 1 h at 4 °C. Subsequently, samples were dehydrated by 10 min washes in 30, 50, 70, and 90% ethanol in water followed by two 100% alcohol washes for 10 min each. Samples were placed in a vacuum oven at room temperature to remove excess alcohol overnight. Then, samples were fixed onto a sample holder, coated with Au for 45 s using a sputter-coater, and imaged using a SEM. The biofilm coverage area was quantified using ImageJ software (shown in Supporting Information, Figure S2). Three locations on the surface of each sample were randomly selected for imaging and used to calculate the biofilm coverage percent as
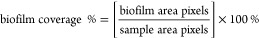


Co-culture biofilms were qualitatively assessed for the presence of rod-shaped *E. coli* and round *S. aureus* (Figure S3 in the Supporting Information).

#### 2.5.5 *Ex Vivo* Wound Infection Model

The 2 h *ex vivo* wound model was modified from the reported methods of Andersson *et al.*(64) Porcine skin was obtained through a tissue sharing program with SUNY Upstate Medical University after receiving institutional approvals. The hair was shaved from the skin sample using a hair clipper. A biopsy punch was used to cut an 8 mm diameter and 2 mm deep round wounds on the surface of pig skin. Subsequently, the pig skin adjacent to the wounds was excised using a surgical scalpel into approximately 12 mm square pieces that fit into the wells of a 12-well plate. The pig skin wounds were disinfected in 70% ethanol for 30 min and air-dried for 2 h in a sterile biosafety cabinet. The porcine skin wound models were then placed into a 12-well plate. Samples were cut into 5 mg pieces (*n* = 3) and sterilized by UV light. Samples were placed in the wounds. Then, 50 μL of *E. coli* or *S. aureus* solutions (OD_600_ = 0.6) were added to wounds to incubate at 37 °C. After 2 h, samples were removed from the wounds using sterile tweezers. The wound was washed twice with 50 μL of sterile PBS, and washings were collected as samples. Samples were serially diluted, drop plated onto LB-agar plates and incubated at 37 °C overnight. CFU images were obtained after 18 h from samples that had been diluted by 10^4^.

The 24 h *ex vivo* wound model was modified from the reported method of Wang and Shukla.^65^ The setup was similar to that described for the 2 h *ex vivo* wound model, except that 3 wounds were cut in each piece of the pig skin, which were filled with samples of the same type (*n* = 3). Then, the pieces of the porcine skin with three wounds were placed in a Petri dish filled with sterile PBS. Samples were cut into 5 mg pieces (*n* = 3), sterilized by UV light, and placed in the wounds. Then, 50 μL of the bacterial solution (OD_600_ = 0.1) was added to each wound, and the skin samples were incubated at 37 °C for 24 h. After 12 h, sterile PBS was added to skin samples to reduce the impacts of solution evaporation. After 24 h, samples were removed from the wounds using sterile tweezers. The wounds were washed twice with 50 μL of sterile PBS, and washings were collected as samples. Samples were serially diluted and drop plated onto LB-agar plates. After incubation at 37 °C for 18 h, CFU images were obtained and analyzed from samples that had been diluted by 10^8^.

### 2.6 Statistics

All statistical analysis was conducted using GraphPad Prism 9. Data were reported as mean ± standard deviation. ANOVA was performed to determine differences between DPCA foams and controls. Statistical significance was taken as ns *p* > 0.05, *p < 0.05, **p < 0.01, ***p < 0.001, and ****p < 0.0001 as compared with AgNP foam and ns *p* > 0.05, #*p* < 0.05, ##*p* < 0.01, ###*p* < 0.001, and ####*p* < 0.0001 as compared with control foam.

## 3 Results and Discussion

### 3.1 DPCA Foam Characterization

**Figure 2:**
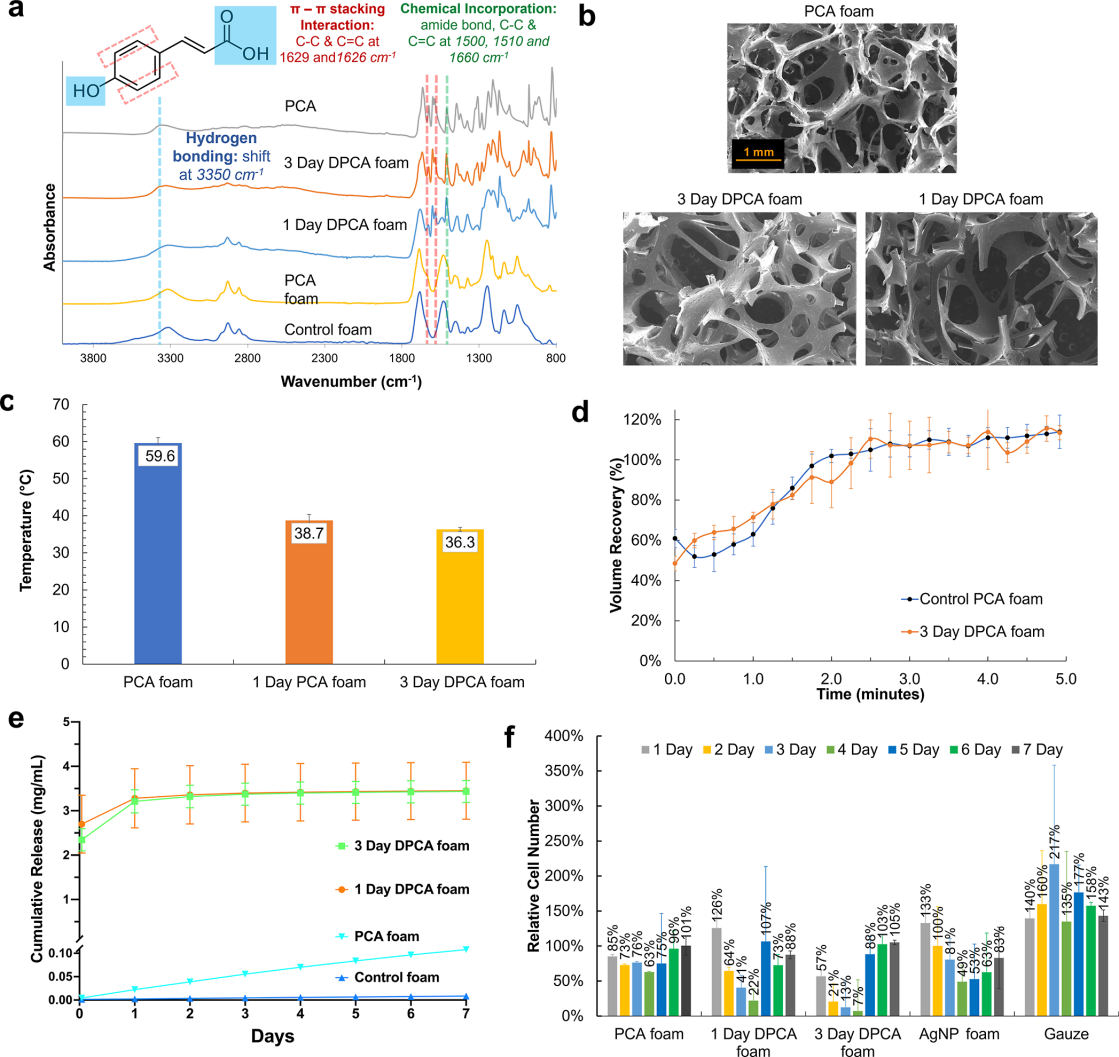
Characterization of DPCA foams. (a) FTIR spectra of synthesized control, PCA (chemical only), 1 day DPCA, and 3 day DPCA foams. (b) SEM images of pore structures of PCA, 1 day DPCA, and 3 day DPCA foams. A scale bar of 1 mm applies to all images. (c) Glass transition temperature (Tg) of PCA, 1 day DPCA, and 3 day DPCA foams measured using differential scanning calorimetry. (d) Volume recovery profile on PCA and 3 day DPCA foams in 37 °C water. (e) PCA cumulative release profile from PCA and DPCA foams over 7 days. (f) 3T3 fibroblast viability measured by relative cell numbers as compared with cells exposed to control foams in the presence of PCA and DPCA foams, AgNP foam, and gauze over 7 days compared with control foam. (c–f) Mean ± standard deviation displayed. *n* = 3.

In Figure 2a, FTIR spectra show that PCA was successfully incorporated into the SMP foam network *via* dual incorporation mechanisms. The amide bond formation at 1500 cm^–1^ indicates that PCA was covalently incorporated (green line). The hydrogen bonds of carboxylic acid and hydroxyl groups (blue line) are shifted from ∼3350 to 3310 cm^–1^.^66^ This shift indicates hydrogen bond formation between PCA molecules that are physically incorporated. Additionally, the 1 day and 3 day DPCA foams showed higher absorbance at 1500 cm^–1^ (C–C bond) and at 1640 cm^–1^ (C=C bond) than PCA foams due to increased concentrations of PCA after physical incorporation. Larger effects were seen in 3 day foams *vs* 1 day foams, indicating that higher loading of physically incorporated PCA occurred during the 3 day incubation. Pulling of the phenolic rings of PCA due to π–π interactions leads to a shift in the C=C bond on the phenolic rings from 1626 to 1629 cm^–1^.^67^ Overall, the observed surface chemistry of DPCA foams indicates successful chemical and physical incorporation of PCA into SMP foams with tunable PCA content based on incubation time frames.

SEM micrographs (Figure 2b) show that DPCA foams have a similar pore structure to PCA foams. Thus, the physical incorporation process did not negatively impact the pore structure of SMP foams. Overall, the pore morphology of all PCA-containing foams was uniform with high interconnectivity.

The Tg of DPCA SMP foams is well above room temperature, as shown in Figure 2c, which enables stable SMP foam storage in the secondary shape. The reduced Tg of DPCA foams relative to PCA foams is attributed to residual DMSO after physical incorporation of PCA, which is corroborated with the observed peak at 1000 cm^–1^ that corresponds with DMSO in FTIR spectra.^68^ In Figure 2d, 3 day DPCA foams achieved 100% volume expansion within ∼2 min in water at 37 °C, with comparable volume recovery profiles to PCA foams. This rapid volume expansion in aqueous environments at body temperature could allow for shape filling of irregularly shaped wounds after implantation. In a hemorrhagic wound, DPCA foams could be inserted in their compressed secondary shapes, after which they would rapidly recover to their expanded primary shape upon heating to body temperature and fill the wound within 2 min to ideally stop bleeding.

Cumulative PCA release profiles demonstrate that PCA exhibits a burst release within 1 h, followed by continuous release of low concentrations of over 7 days from both DPCA foams, as shown in Figure 2e. DPCA foams release approximately 2.5 mg/mL of PCA within 1 h. This concentration is higher than the reported minimum inhibitory concentration (MIC), minimum bactericidal concentration (MBC), and minimal biofilm inhibitory concentration (MBIC) of PCA against multiple bacteria (e.g., 0.02 mg/mL MIC for *S. aureus*,^58^ 0.08 mg/mL MIC for *E. coli*,^58^ 1 mg/mL MBC for *Salmonella enteritidis*,^69^ and 0.25 mg/mL MBIC for *S. enteritidis*(69)). The cumulative released PCA concentration reaches 3.2 mg/mL within 24 h. After that, the release of PCA slows down, but the PCA concentration continues to rise slowly over the full 7 days. The chemically incorporated PCA foams exhibit a slow linear release of PCA over the full 7 days, which corresponds with the slower release from DPCA foams between days 1 and 7.

The density of 3T3 fibroblasts was characterized over 7 days of incubation with samples relative to controls, as shown in Figure 2f. Initial cell numbers were affected by high concentrations of released PCA. In the first 24 h, cell numbers in the presence of PCA foams, 1 day DPCA foams, and 3 day DPCA foams were 85, 128, and 52%, respectively, in comparison with cells in the presence of control foams. After 24 h, the relative cell density started to decline to 73, 64, and 21% for PCA, 1 day DPCA, and 3 day DPCA foams, respectively. Relative cell numbers continued to decline over days 3 and 4 in all PCA-containing foams, with larger effects seen in 3 day DPCA foams. However, after 5 days, cell density returned to a high level (≥75%) in the presence of all PCA-containing foams for the remainder of the 7 day experiment. We hypothesize that these effects were due to the burst release of physically incorporated PCA, which inhibited cell proliferation during the first 5 days. Qualitatively, cells did not look like they were dying at early time points but instead had lower density due to decreased proliferation. At later time points, PCA release was at lower concentrations, allowing cell proliferation to return to normal levels. These results correlate well with studies on phenolic acids as anticancer agents based on their ability to inhibit cell proliferation.^45−48^

There are some discrepancies between the release profiles in Figure 2e and the cytocompatibility data (e.g., higher viability at 24 h and larger differences between 1 day and 3 day DPCA foams), which we attribute to reduced PCA solubility in aqueous cell culture media as compared with the PBS/DMSO solutions used for release profiles. PCA is not highly water soluble and, therefore likely exhibits a delay in the initial release in media. When using DPCA foams as hemostatic agents, clinicians could change the DPCA foam within 24 h of hospitalization to improve cytocompatibility and enhance healing. Interestingly, the AgNP foam had high initial cell viability (133% in comparison with control foams) that also reduced over time. Between days 4 and 6, viability in the presence of AgNP foams was 49–63%, with observed increases to 83% viability at day 7. We hypothesize that time points with lower viability likely correspond with higher concentrations of released AgNPs from these dressings.

### 3.2 Antimicrobial Testing: Single-Species Bacteria

**Figure 3:**
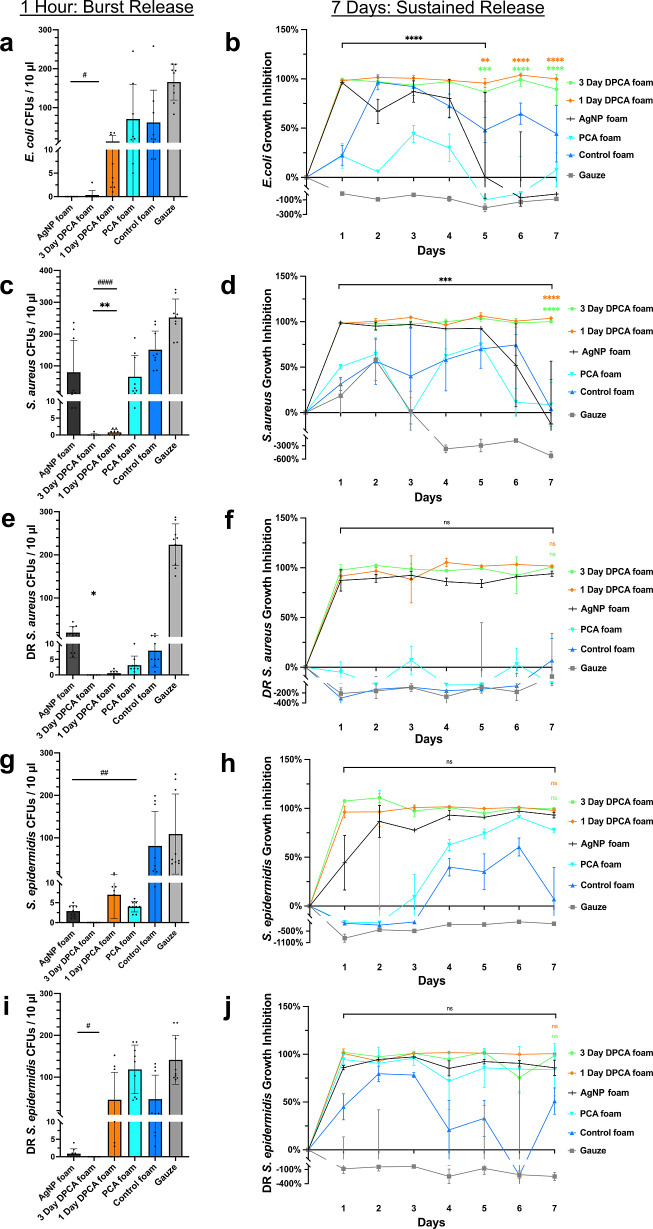
Initial and sustained antimicrobial properties of DPCA foams at 1 h and over 7 days. *E. coli* at (a) 1 h and (b) over 7 days. *S. aureus* at (c) 1 h and (d) over 7 days. Drug-resistant (DR) *S. aureus* at (e) 1 h and (f) over 7 days. *S. epidermidis* at (g) 1 h and (h) over 7 days. DR *S. epidermidis* at (i) 1 h and (j) over 7 days. Mean ± standard deviation displayed. *n* = 3. ns: *p* > 0.05; *p < 0.05, **p < 0.01, ***p < 0.001, and ****p < 0.0001 as compared with AgNP foam. #*p* < 0.05, ##*p* < 0.01, and ####*p* < 0.0001 as compared with control foam.

Figure 3a,b shows that the PCA in 3 day DPCA foams was effective at eliminating the majority of *E. coli* CFUs after 1 h of incubation at levels that were comparable to the AgNP foam clinical control. Additionally, 3 day DPCA foams had higher efficacy in comparison with both 1 day DPCA and PCA foams. In the 7 day antimicrobial testing, both 1 and 3 day DPCA foams inhibited 100% of *E. coli* growth starting at 1 day of testing. AgNP foams had high initial effects against *E. coli* at 1 h and through 3 days, but the growth inhibition efficacy significantly decreased after the 4th day of testing.

In Figure 3c,d, 1 day and 3 day DPCA foams showed improved initial (1 h) antimicrobial properties against *S. aureus* compared with AgNP foams and PCA foams, which had similar efficacy. However, after 1 day of incubation, the AgNP foam and DPCA foams had comparably high growth inhibition at levels that were higher than that of the PCA foam. After the fifth day, the *S. aureus* growth inhibition rate decreased in the presence of AgNP foams, while DPCA foams retained their 100% growth inhibition over the full 7 days of testing. For drug-resistant *S. aureus*, 1 and 3 day DPCA foams demonstrated excellent antimicrobial properties compared with AgNP foam and control foams, as shown in Figure 3e. Interestingly, DPCA foams and AgNP foam inhibited 100% of the growth of DR *S. aureus* over 7 days, Figure 3f. In recent studies,^70^ AgNPs showed sustainable antimicrobial effects against DR *S. aureus* through disruption of key proteins. AgNPs are more effective than antibiotics (*e.g.*, ampicillin) against drug-resistant bacteria. These results may explain why the AgNPs foam was more effective against DR *S. aureus* in comparison with the native strain in our studies.

In Figure 3g,h, the 3 day DPCA foam killed almost all *S. epidermidis* within 1 h, while AgNP foams, 1 day DPCA foams, and PCA foams had significantly higher CFUs. In 7 day antimicrobial testing, 1 and 3 day DPCA foams and AgNP foams presented similarly high *S. epidermidis* inhibition. Similar trends were observed with DR *S. epidermidis* in Figure 3i,j.

In general, in the 7 day antimicrobial testing, DPCA foams proved to continuously inhibit the growth of three strains of native bacteria (Gram positive and negative) and two strains of drug-resistant bacteria. This result is attributed to the continual release and/or presentation of PCA from DPCA foams through the unique dual-release mechanism, as shown in Figure 1b, and corresponds with the PCA release profiles shown in Figure 2e. DPCA foams had better longer term antimicrobial protection against *E. coli* and *S. aureus* than the AgNP foam. According to Kędziora et al.,^71^ AgNPs alter the susceptibility of *E. coli* and *S. aureus* strains after 6 days of exposure, and their MIC increases over time. The combination of AgNPs with antibiotics has been historically thought to improve antibiotic efficacy. However, in this prior study, it was also found that bacterial antibiotic susceptibility was reduced after prolonged exposure to AgNPs. Therefore, AgNPs are not appropriate antimicrobial agents for preventing wound infections in the long term.

In contrast, previous work showed that PCA does not induce resistance in *S. aureus* over 6 days of continuous exposure, which supports the results shown here.^72^ Therefore, PCA has the potential to be suitable for long-term antimicrobial protection. DPCA foams also showed better short-time protection than AgNP foam for native and drug-resistant bacteria. As confirmed in our previous study and validated here, PCA chemically incorporated into the polyurethane network will be more stable and exhibit a sustained release from the network.^18^ We envision that DPCA foams could be used by medical care providers for trauma wound treatment and care to reduce wound infection before and during hospitalization. The longer antimicrobial persistence could also enable a reduction in dressing change frequency, thereby lowering pain and improving patient compliance. These antimicrobial effects would have to be balanced with the cell viability changes over time in a finalized treatment plan.

### 3.3 Antimicrobial Testing: Mixed-Species Bacteria

**Figure 4:**
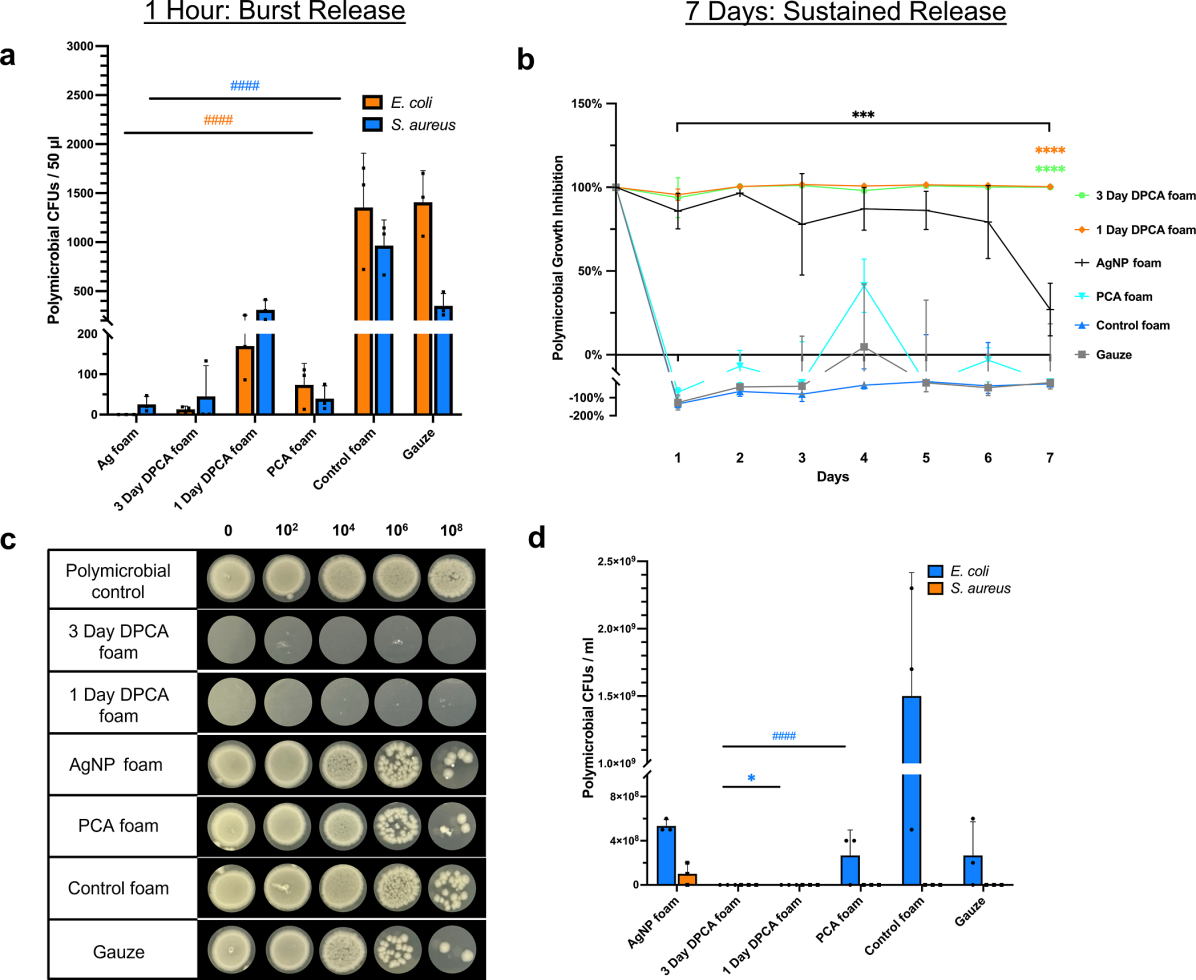
Initial and sustained antimicrobial properties of DPCA foams in co-culture of *E. coli* and *S. aureus* at 1 h and over 7 days. (a) Polymicrobial colony formation unit (CFU) densities after 1 h incubation with SMP foams. (b) Optical density (OD) of polymicrobial solution in the presence of DPCA foams over 7 days. (c) Polymicrobial colony formation after 7 days of incubation with samples. (d) Polymicrobial CFU density quantification at 7 days. Mean ± standard deviation displayed. *n* = 3. *p < 0.05, ***p < 0.001, and ****p < 0.0001 as compared with AgNP foam. ####*p* < 0.0001 as compared with control foam.

In Figure 4a, the 3 day DPCA foam and PCA foam significantly reduced 95% of polymicrobial CFUs (*E. coli* and *S. aureus*) compared with the control foam within 1 h to levels that were statistically comparable to the AgNP clinical control. The 3 day and 1 day DPCA foams also inhibited 100% of polymicrobial growth over 7 days, while the polymicrobial growth inhibition rate of AgNP foams decreased after 6 days of incubation, as shown in Figure 4b. Figure 4c shows complete elimination of polymicrobial CFUs on day 7 after incubation with both DPCA foams, while all other samples had full coverage of droplet areas with a “lawn” of bacteria at dilutions between 0 and 10^4^. When quantifying these CFUs, as shown in Figure 4d, it is apparent that the observed reductions in bacterial density were significant after DPCA foam exposure in comparison with all other groups.

These results show effective initial and sustained inhibition of *E. coli* and *S. aureus* co-cultures by DPCA foams over up to 7 days. On day 7, *E. coli* ATCC 9637 became the dominant species among the polymicrobial cultures with all samples, as indicated by the larger colonies (*vs* smaller area colonies associated with *S. aureus* ATCC 51153), as shown in Figure 4c. It is important to note that polymicrobial cultures grow faster than single bacteria, and polymicrobial infections have a higher tolerance to antiseptics as compared with single species bacteria.^73^ Wounds with multi-bacterial infections are more challenging to heal due to interactions between bacteria, which can include the exchange of drug-resistant genes.^74^ Excellent antimicrobial properties of PCA-containing foams against mixed species bacteria with complete elimination at 7 days is very promising for their potential use as antimicrobial hemostatic wound dressings to reduce infection risks.

### 3.4 Anti-Biofilm Properties

**Figure 5:**
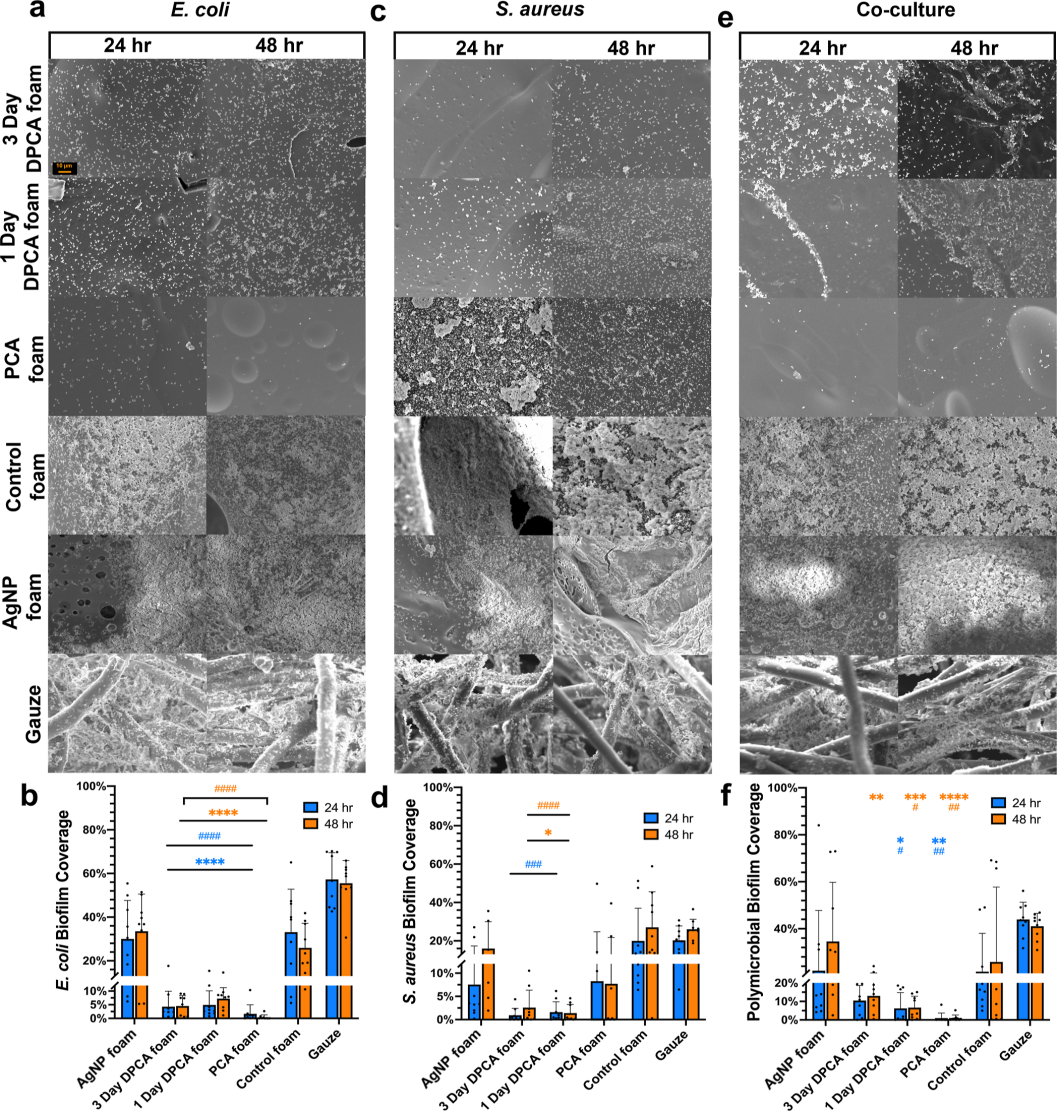
Qualitative and quantitative characterization of *E. coli*, *S. aureus,* and polymicrobial biofilm coverage on the surface of SMP foams and clinical controls. Scanning electron micrographs (1000X) of (a) *E. coli* biofilm with (b) quantified *E. coli* biofilm coverage percent; (c) *S. aureus* biofilm with (d) quantified *S. aureus* biofilm coverage; and (e) co-culture of *E. coli* and *S. aureus* biofilm with (f) quantified co-culture biofilm coverage. Mean ± standard deviation displayed. *n* = 3. *p < 0.05, **p < 0.01, ***p < 0.001, and ****p < 0.0001 as compared with AgNP foam. #*p* < 0.05, ##*p* < 0.01, ###*p* < 0.001, and ####*p* < 0.0001 as compared with control foam. Scale bar of 10 μm applies to all images.

For *E. coli* biofilm formation, the mean coverage area on all PCA-containing foams was below 7% at 24 h and 48 h, as shown in Figure 5a,b, with particularly low biofilm coverage on PCA foams, at approximately 0.4% at 24 h and 1.7% at 48 h. The mean biofilm coverage on AgNP and control foams was significantly higher, reaching approximately 30% at 24 and 58 h, and biofilm coverage was the highest on gauze, at 56% after 24 and 48 h of incubation.

As shown in Figure 5c,d, the mean coverage of *S. aureus* biofilms was below 3% on 1 day and 3 day DPCA foams at both 24 and 48 h. Biofilm coverage on PCA foams was higher with *S. aureus* as compared with *E. coli*, reaching approximately 8% at 24 and 48 h. AgNP foams had significantly higher biofilm coverage as compared with DPCA foams, with ∼8% coverage at 24 h and 16% coverage at 48 h. Control foams and gauze had a similarly high biofilm coverage (∼20–30%) at both time points.

Figure 5e,f shows that the mean biofilm coverage after co-culture with *E. coli* and *S. aureus* was the lowest on PCA foams, at approximately 1% at 24 and 48 h. Polymicrobial biofilm coverage on DPCA foams ranged from ∼5 to 11%. The coverage on AgNPs and control foams were significantly higher, at approximately 22% at 24 h and 33–35% at 48 h for both materials. Gauze had the highest polymicrobial biofilm coverage of 42% at 24 and 48 h.

**Figure 6:**
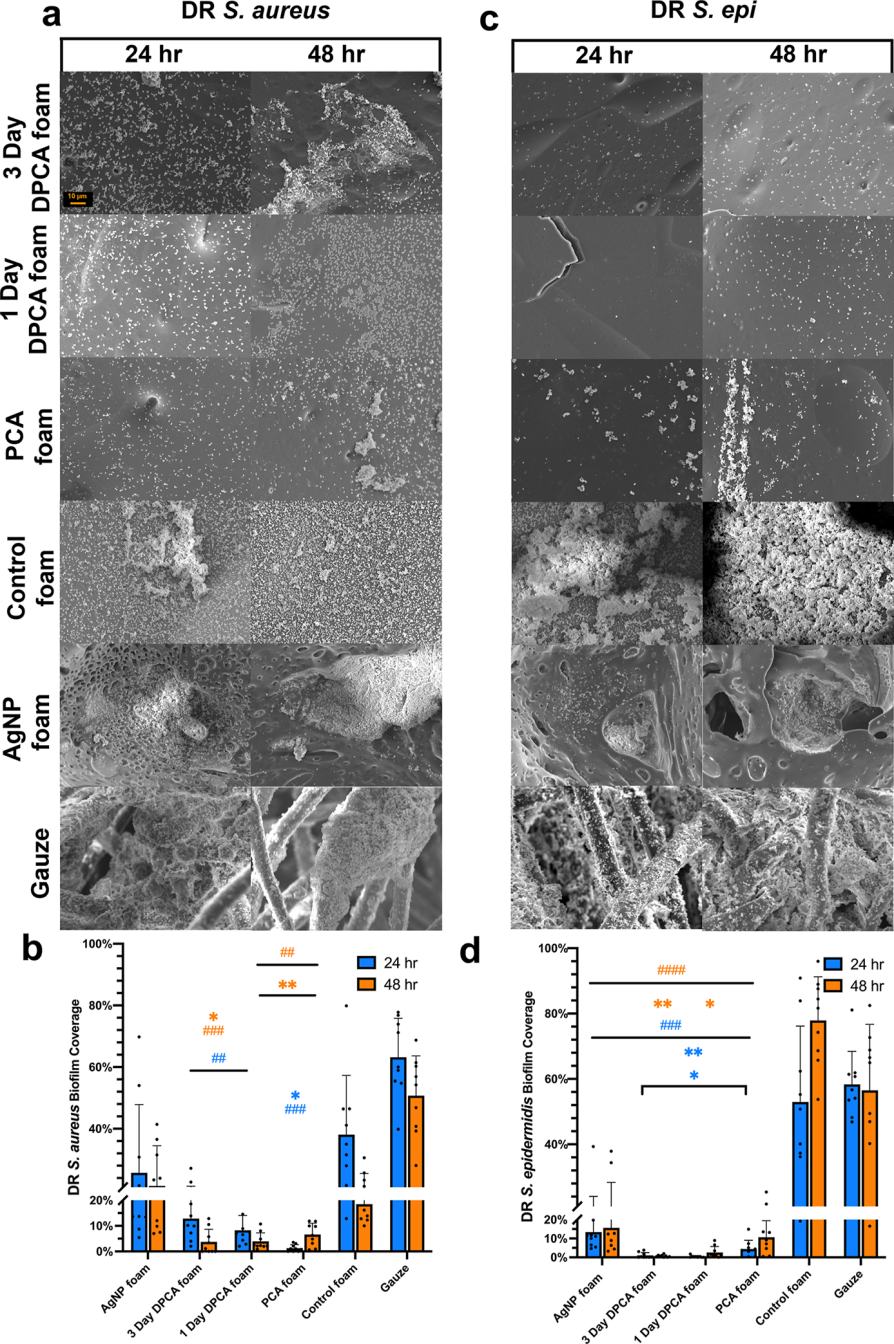
Qualitative and quantitative characterization of drug-resistant (DR) *S. aureus* and *S. epidermidis*. biofilm coverage on the surface of SMP foams and clinical controls. Scanning electron micrographs (1000X) of (a) DR *S. aureus* biofilm with (b) quantified DR *S. aureus* biofilm coverage percent and (c) DR *S. epidermidis.* biofilm with (d) quantified DR *S. epidermidis.* biofilm coverage. Mean ± standard deviation displayed. *n* = 3. *p < 0.05 and **p < 0.01 as compared with AgNP foam. ##*p* < 0.01, ###*p* < 0.001, and ####*p* < 0.0001 as compared with control foam. Scale bar of 10 μm applies to all images.

DR bacterial biofilms are shown in Figure 6a,b. The mean biofilm coverage of DR *S. aureus* was 13% for 3 day DPCA foams, 8% for 1 day DPCA foams, and 1% for PCA foams at 24 h. After 48 h, the mean coverage of DR *S. aureus* biofilm was reduced to below 4% on 1 day and 3 day DPCA foams, and the mean coverage on PCA foams was increased to 7%. The mean DR *S. aureus* biofilm coverage on AgNP foam was between 21 and 26% over the two time points. The control foam had a higher biofilm coverage at 24 h (38%) that was reduced to 19% by 48 h. For gauze, the biofilm coverage was 51–63%.

As shown in Figure 6c,d, the mean coverage of DR *S. epidermidis* biofilm on 1 and 3 day DPCA foams was below 3% at 24 and 48 h. The mean coverages of DR *S. epidermidis* biofilms on PCA foams were higher at 4 and 11% at 24 and 48 h, respectively. The mean biofilm coverage on AgNP foams was constant at approximately 15% at 24 and 48 h, and the biofilm coverage on gauze was approximately 58% at 24 and 48 h.

In the biofilm testing, we employed different strains of bacteria that display two main biofilm formation mechanisms: *E. coli* is a motile organism that easily forms biofilms at the air–liquid interface,^75^ while *S. aureus* is a non-motile organism that forms biofilms on the surface of materials. The chemically incorporated PCA within SMP foams can effectivity inhibit biofilm generation on the surface. Despite having lower overall concentrations of PCA, PCA foam showed the lowest biofilm coverage in the co-culture with both cell types. We hypothesize that this effect may be attributed to reduced PCA antimicrobial activity in DPCA foams due to interactions between physically and chemically incorporated PCA. By participating in hydrogen bonding and/or π–π stacking, the active antimicrobial groups on these PCA molecules may be hidden or less available on the surface in DPCA foams.^76^

The final phase of biofilm development is the dispersion phase. In this phase, planktonic cells are released from the biofilm to start a new biofilm life cycle.^77^ Non-antibiofilm wound dressings act as incubators to release new planktonic cells after the surface biofilm matures; for example, in the biofilm assays shown here, the gauze clinical control showed a high biofilm coverage with all tested bacteria strains. Therefore, wound dressings that are designed to reduce infection risks should minimize initial bacteria attachment and kill released bacteria during the dispersion phase.

**Figure 7:**
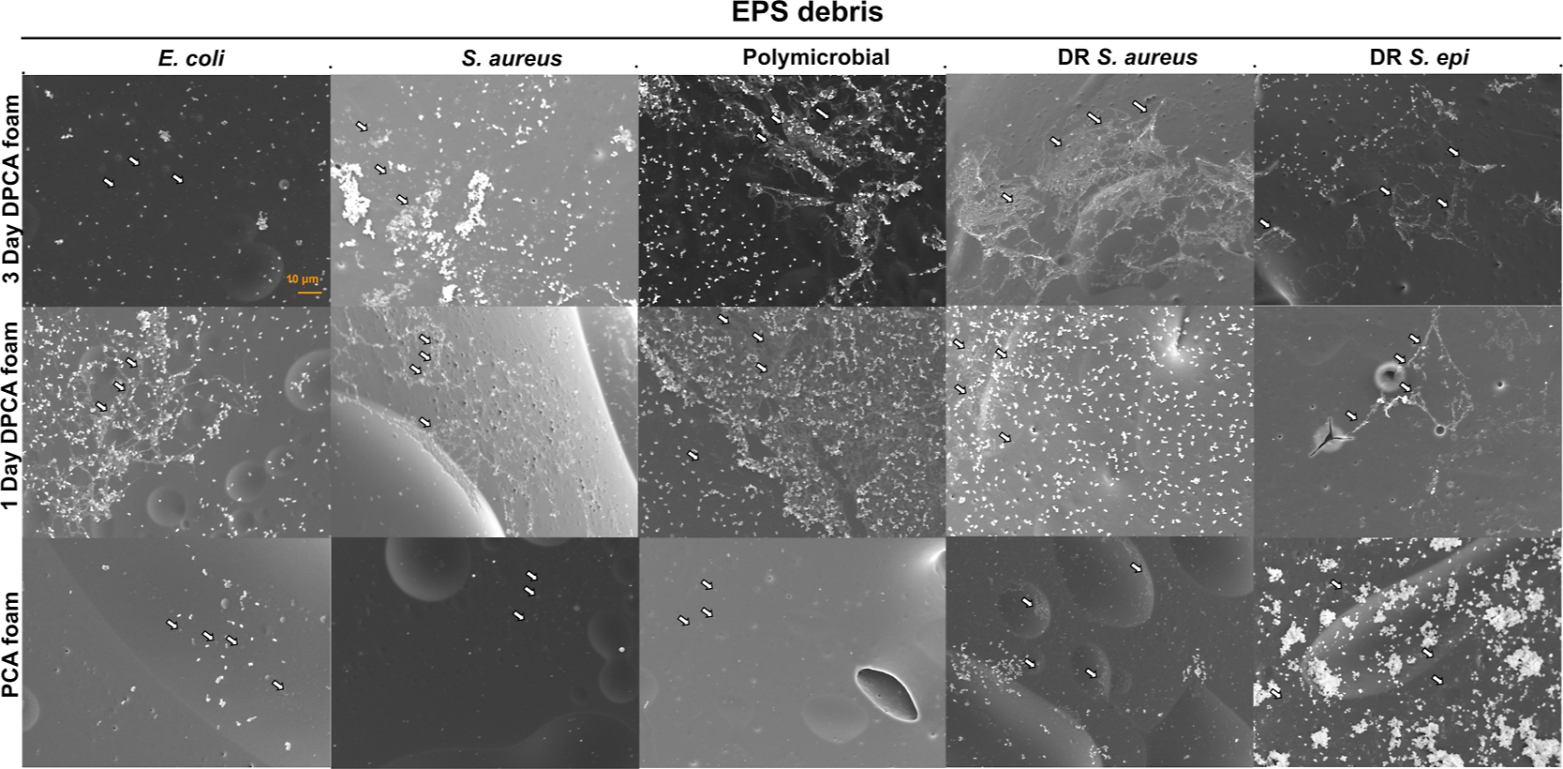
Scanning electron micrographs (1000X) showing EPS debris observed on the surface of DPCA and PCA foams after 48 h in the biofilm formation study. A scale bar of 10 μm applies to all images.

When evaluating the observed decreases in the biofilm coverage between 24 and 48 h, additional structures were noted on several sample surfaces. In Figure 7, the white arrows indicate extracellular polymeric substance (EPS) debris from biofilms on the surfaces of all PCA-containing foams with all tested bacteria strains (*E. coli*, *S. aureus*, polymicrobial, DR *S. aureus*, and DR *S. epidermidis*.). These EPS debris are typically visible upon bacteria disintegration and death,^78^ indicating that bacteria may have initially attached to PCA-containing foam surfaces but later died due to interactions with incorporated PCA.

We also found that the morphology of *E. coli* became shorter on the surface of DPCA foams in comparison with *E. coli* on control foams (Supporting Information, Figure S4). Namely, *E. coli* changed from the original rod shape to a more spherical shape, Figure 6a. In Matuła *et al.*,^79^ it was observed that *E. coli* undergoes phenotypic changes in the face of survival stress as a sign of distress. This observation indicates that *E. coli* may enter a viable but non-culturable (VBNC) state after exposure to DPCA foams, where bacteria cannot regain cultivability. Further studies are needed to learn more about these effects and to determine whether other tested strains are morphologically altered.

Overall, these results demonstrate that PCA can inhibit the formation of many types of biofilm, including drug-resistant bacterial biofilms. By continuously releasing PCA from DPCA foams, these materials could prevent biofilm formation in the wound to help promote healing.

### 3.5 *Ex Vivo* Infection Control

**Figure 8:**
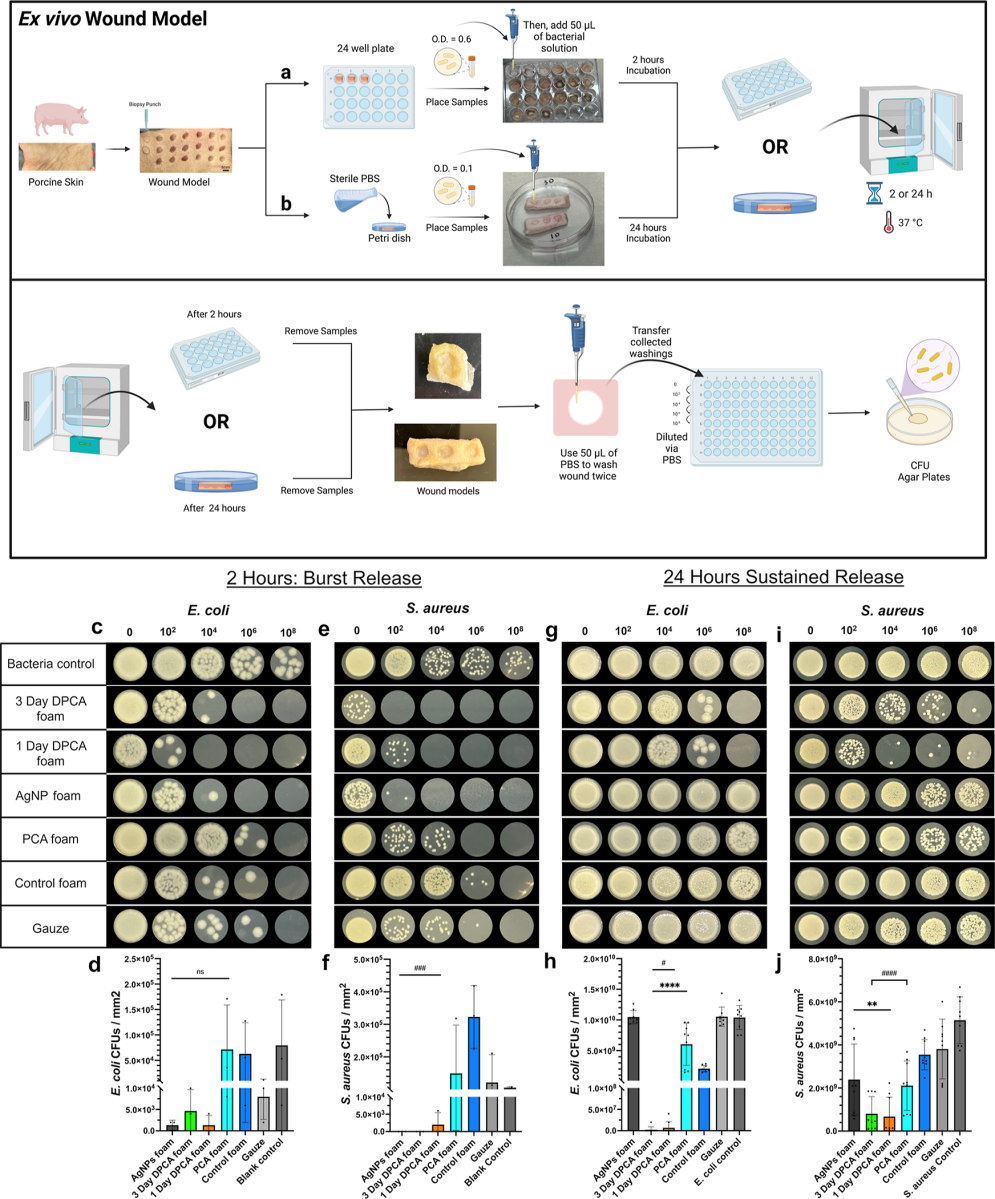
Schematic representation of characterization of antimicrobial properties in an *ex vivo* porcine wound model and results for *E. coli* and *S. aureus* in the wound model at 2 and 24 h. Schematics of experimental process of antimicrobial testing in the *ex vivo* wound model over (a) 2 and (b) 24 h. Created with BioRender.com. (c) Images of *E. coli* colony forming units (CFUs) after 2 h with (d) quantified *E. coli* CFUs. (e) Images of *S. aureus* CFUs after 2 h with (f) quantified *S. aureus* CFUs. (g) Images of *E. coli* CFUs after 24 h with (h) quantified *E. coli* CFUs. (i) Images of *S. aureus* CFUs after 24 h with (j) quantified *S. aureus* CFUs. Mean ± standard deviation displayed. *n* = 3. ns: not significant. **p < 0.01 and ****p < 0.0001 as compared with AgNP foam. #*p* < 0.05 and ####*p* < 0.0001 as compared with control foam. A scale bar of 10 μm applies to all images.

Foams were characterized in an *ex vivo* model of skin wound infection for 2 and 24 h (Figure 8a,b). The 1 and 3 day DPCA foams and AgNP foam reduced 94–98% of *E. coli* CFUs as compared with the control group in *ex vivo* porcine skin wounds at 2 h, as shown in Figure 8c,d. For *S. aureus* infected *ex vivo* wounds, as shown in Figure 8e,f, the AgNP foam and 3 day DPCA foam reduced 98–100% of CFUs within 2 h, while the 1 day DPCA foam decreased CFUs by ∼98%. In Figure 8g,h, it can be seen that the AgNP and PCA foams decreased *E. coli* CFUs by approximately 99.9%, while Figure 8i,j shows that the 1 day and 3 day DPCA foams reduced 84–87% of *S. aureus* CFUs in the wound after 24 h.

In general, the DPCA foams demonstrated similar results *ex vivo* to those measured *in vitro*. Overall, DPCA foams significantly reduce the number of bacteria in wounds and are effective against both Gram-negative and positive strains. We hypothesize that the physically incorporated PCA is burst released from DPCA foams to rapidly kill high concentrations of bacteria in the wound, while more tightly bound physically incorporated PCA and covalently bound chemically incorporated PCA provide sustained effects over longer time frames.

## 4 Conclusions

In conclusion, DPCA foams exhibit excellent antimicrobial and anti-biofilm properties against native and drug-resistant strains, Gram-positive and Gram-negative bacteria, and single and mixed species cultures. Furthermore, the dual incorporation mechanism enables loading of PCA with multiple types of interactions into synthetic SMP foams. Chemically incorporated PCA is covalently tethered *via* amide groups to provide long-term antimicrobial properties. PCA in the polyurethane network can effectively prevent biofilm formation and bacterial attachment to the surface. Physically incorporated PCA can exhibit both a burst and sustained release from DPCA foams over at least 7 days, based on variations between physical bond strength with hydrogen bonding and π–π interactions. Notably, burst release of PCA from DPCA foams within 1 h kills bacteria in high concentrations, and DPCA foams demonstrate 100% growth inhibition for 3 native strains, 2 drug-resistant strains, and polymicrobial strains over 7 days of culture. *Ex vivo* antimicrobial testing provides an initial indication of clinical feasibility and showed similarly effective antimicrobial properties to those observed in *in vitro* studies.

In addition to antimicrobial and anti-biofilm properties, DPCA foams maintain the required physical and shape memory properties for their potential application as hemostatic dressings. DPCA foams exhibit good shape memory properties and can expand from a compressed temporary shape to an expanded primary shape within 2 min. DPCA foams also showed generally good cytocompatibility over 7 days. In the long term, DPCA foams could provide an easy-to-use hemostatic agent that can be “injected” directly into a bleeding wound, where they disinfect the wound as they stop bleeding to reduce wound infection risks. DPCA foams could also be used as a wound dressing to prevent hospital-acquired infections and drug-resistant bacterial infections while the patient is recovering from traumatic injury.

## Supporting Information Available

Method for CFU quantification using ImageJ software; method for biofilm coverage quantification using ImageJ software; scanning electron micrographs of co-culture biofilms used for the qualitative assessment of the presence of rod-shaped *E. coli* and round *S. aureus*; and scanning electron micrographs showing the morphology of *E. coli* from the rod to round shape on DPCA foams (PDF)
