# Supplementary material for: Dual Burst and Sustained Release of p-Coumaric Acid from Shape Memory Polymer Foams for Polymicrobial Infection Prevention in Trauma-Related Hemorrhagic Wounds
Source: ACS Appl Mater Interfaces. 2023 May 15;15(20):24228–43. doi: 10.1021/acsami.3c04392 (PMC10214375; doi:10.1021/acsami.3c04392)
Supplement: Supplementary file 1 — am3c04392_si_001.pdf [file am3c04392_si_001.pdf]

Supporting Information

Dual Burst and Sustained Release of p-Coumaric Acid from Shape Memory Polymer Foams for Polymicrobial Infection Prevention in Trauma-related Hemorrhagic Wounds

Changling Du,[1] David Anthony Fikhman[1], Devanand Persaud[1] and Mary Beth Browning Monroe[1*]

[1]Department of Biomedical and Chemical Engineering, Bioinspired Institute for Material and Living Systems, Syracuse University, Syracuse, NY, 13244, U.S.A.

*Corresponding author:
Dr. Mary Beth Browning Monroe
Department of Biomedical and Chemical Engineering
Bioinspired Institute for Material and Living Systems
Syracuse University
318 Bowne Hall
Syracuse, NY 13244
Tel: (315) 443-3323
E-mail: mbmonroe@syr.edu

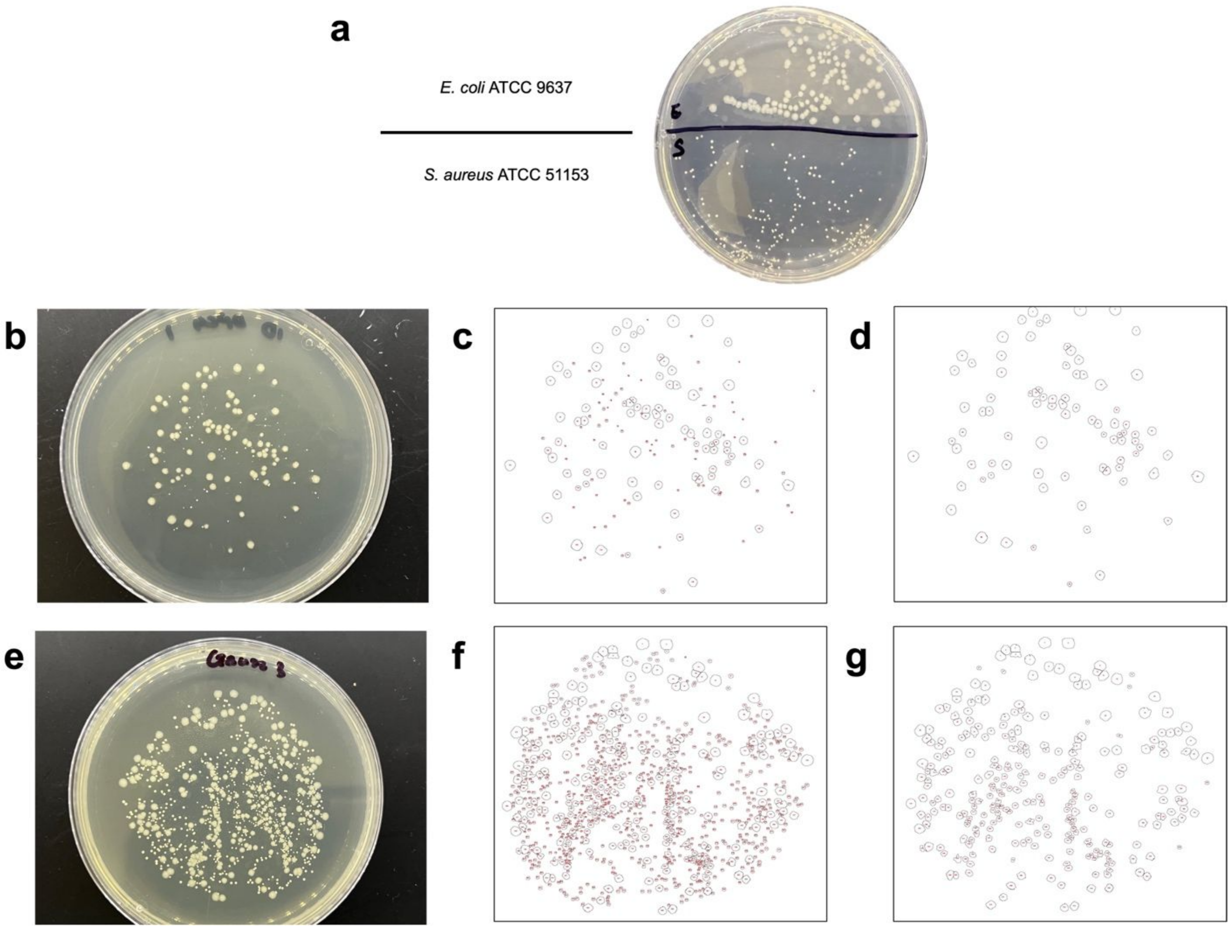

Figure S1. a. Colonies of *E. coli* ATCC 9637 (Top) and *S. aureus* ATCC 51153 (Bottom) on LB agar plates. **b.** Colonies of co-culture of *E. coli* and *S. aureus* after incubation with control foam. **c.** Colonies of *E. coli* and *S. aureus* pictures processed by ImageJ. **d.** Smaller colonies of *E. coli* were screened out from larger *S. aureus* colonies via ImageJ processing to quantify types of colonies. **e.** Colonies of co-culture of *E. coli* and *S. aureus* after incubation with gauze. **f.** Colonies of *E. coli* and *S. aureus* pictures processed by ImageJ. **g.** Smaller colonies of *E. coli* were screened out from larger *S. aureus* colonies via ImageJ processing to quantify types of colonies.

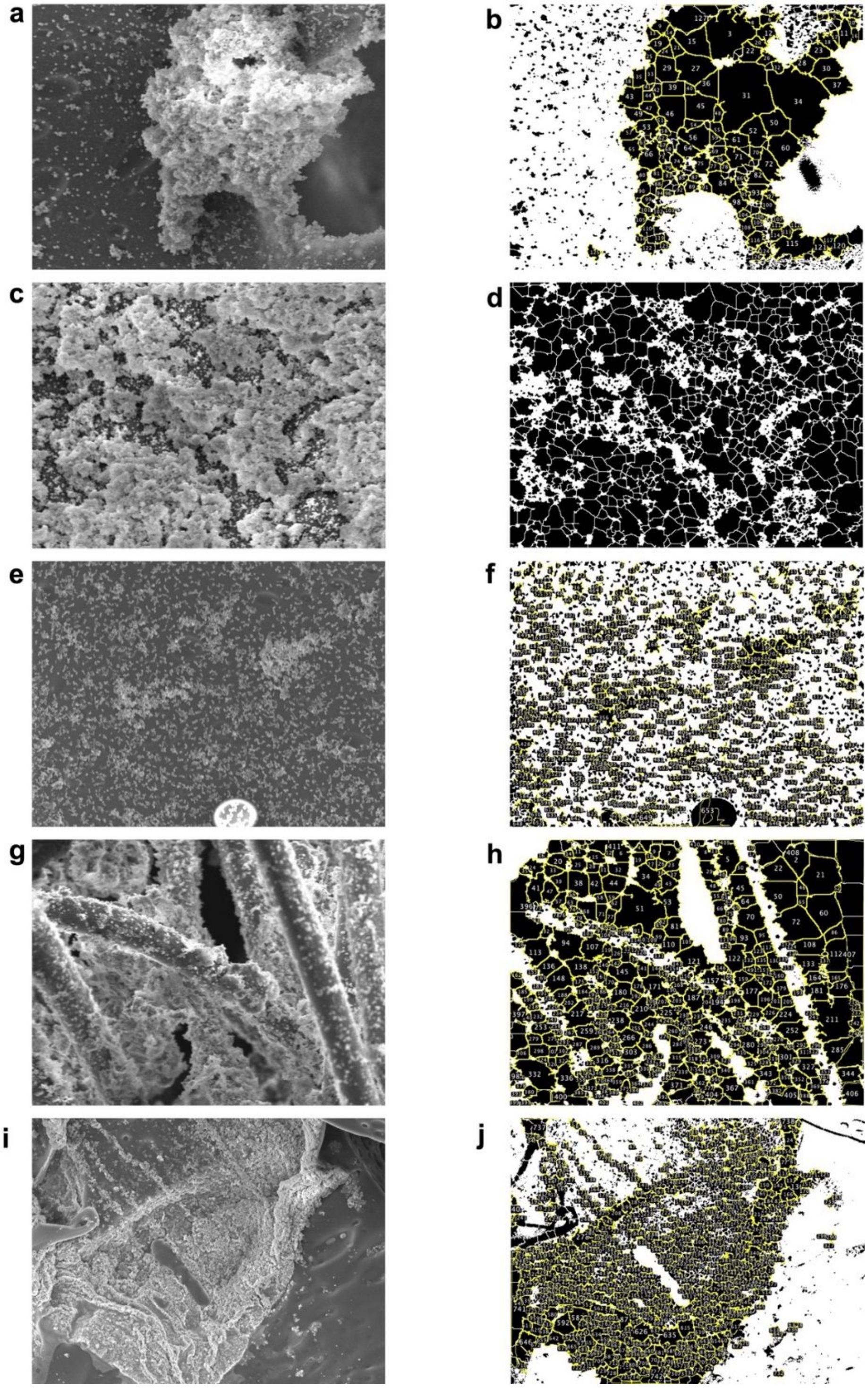

Figure S2. a. *S. aureus* SEM micrograph of biofilm on the surface of control foam **b.** *S. aureus* SEM image of biofilm after ImageJ processing. **c.** *S. aureus* SEM micrograph of biofilm on the surface of control foam **d.** *S. aureus* SEM image of biofilm after ImageJ processing. **e.** *E. coli* SEM micrograph of biofilm on the surface of control foam **f.** *E. coli* SEM image of biofilm after ImageJ processing. **g.** DR. *S. epidermidis* SEM micrograph of biofilm on the surface of gauze **h.** DR. *S. epidermidis* SEM image of biofilm after ImageJ processing. **i.** DR. *S. epidermidis* SEM micrograph of biofilm on the surface of AgNPs foam **j.** DR. *S. epidermidis* SEM image of biofilm after ImageJ processing.

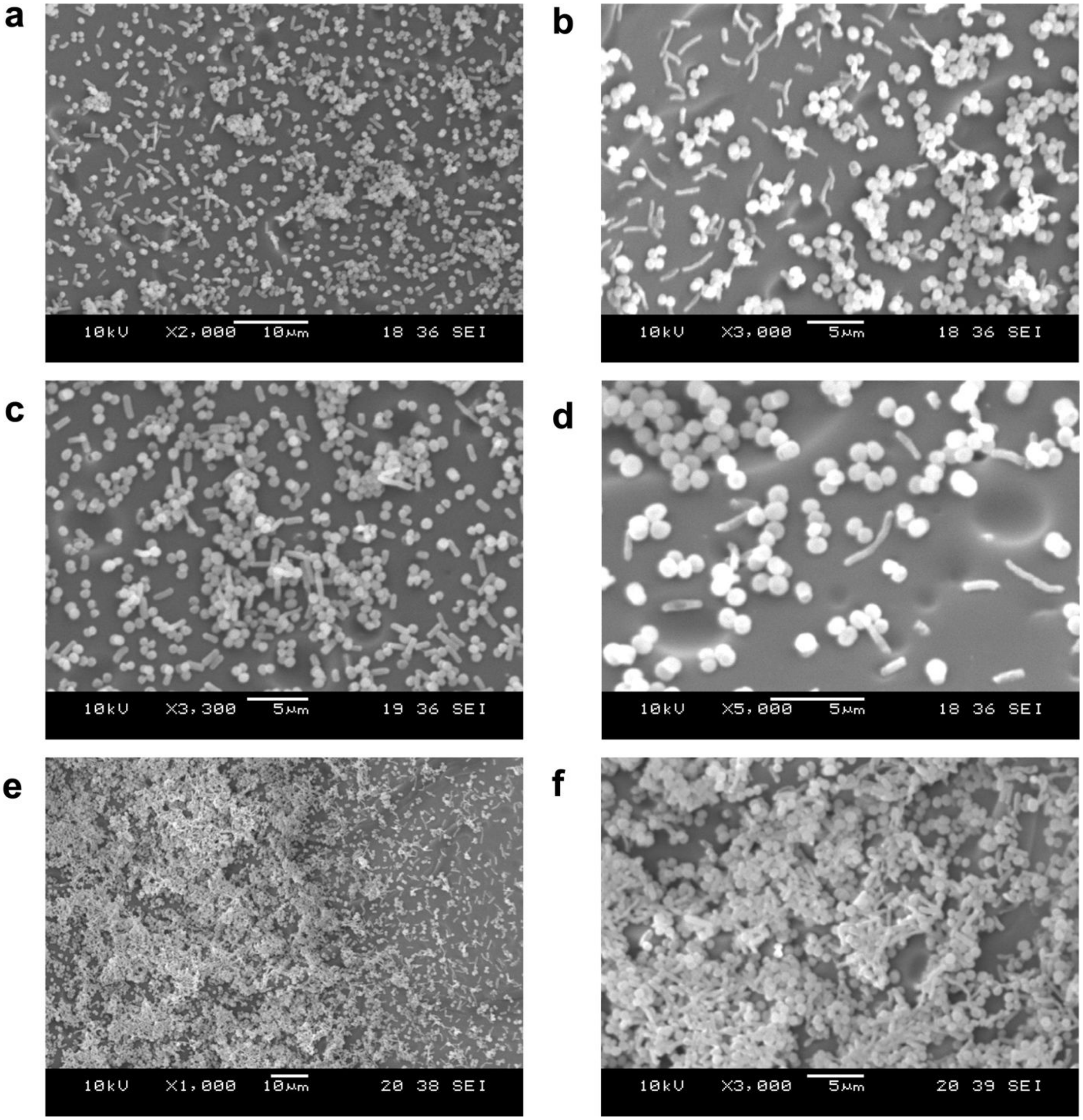

Figure S3. a. SEM micrographs of co-cultured *E. coli* and *S. aureus* biofilms on the surface of control foams after 24 hours incubation. 1000 X magnification **b.** 3000 X magnification. **c.** 3300 X magnification. **d.** 5000 X magnification. **e.** SEM micrograph of co-cultured *E. coli* and *S. aureus* multi-layer biofilms on the surface of control foams after 24 hours incubation. 1000 X magnification **f.** 3000 X magnification

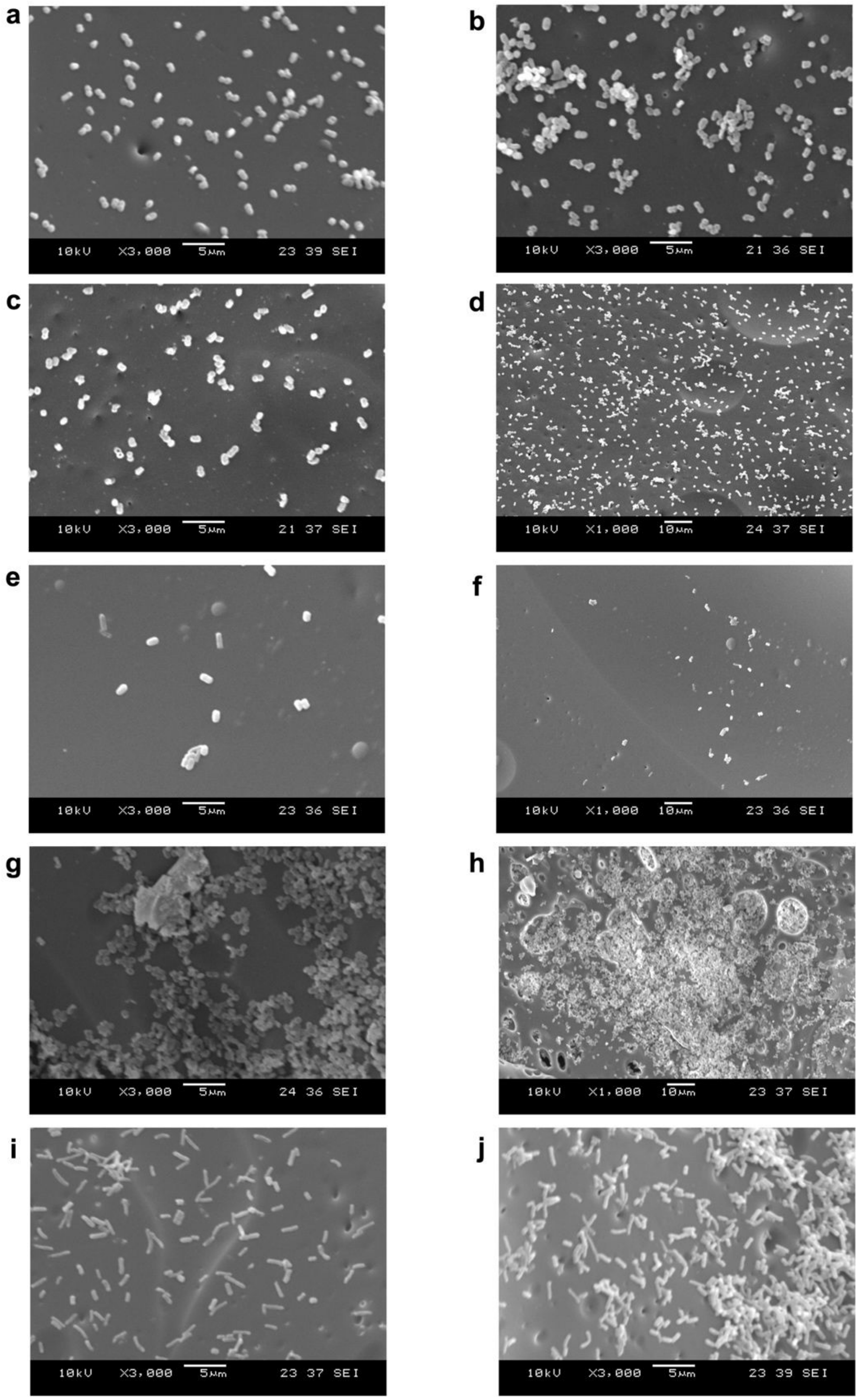

Figure S4. a. SEM micrographs of *E. coli* biofilms on the surface of 3 Day DPCA foam. 1000 X magnification. **b.** 3000 X magnification. **c.** *E. coli* biofilms on the surface of 1 Day DPCA foam. 3000 X magnification. **d.** 1000 X magnification. **e.** *E. coli* biofilms on the surface of PCA foam. 3000 X magnification. **f.** 1000 X magnification. **g.** *E. coli* biofilms on the surface of AgNPs foam. 3000 X magnification. **h.** 1000 X magnification. **i-j.** *E. coli* biofilms on the surface of control foam. 3000 X magnification.
